# Short-term outcomes of a micro-choice-based intervention for chronic low back pain: a quasi-experimental study

**DOI:** 10.1186/s12891-026-09635-x

**Published:** 2026-02-17

**Authors:** Christoffer Børsheim, Silje Mæland, Eirik Søfteland, Sigurd W. Hystad, Gerd Kvale, Andreas Tunset, Øystein Ødegaard-Olsen, Jan Hartvigsen, Thomas Kadar

**Affiliations:** 1 Department of Global Public Health and Primary Care, University of Bergen, Bergen, Norway; 2 Department of Psychosocial Science, University of Bergen, Bergen, Norway; 3 Department of Clinical Psychology, University of Bergen, Bergen, Norway; 4 Department of Clinical Medicine, University of Bergen, Bergen, Norway; 5 Department of Physical Medicine and Rehabilitation, Haukeland University Hospital, Bergen, Norway; 6 Division of Mental Health, Haukeland University Hospital, Bergen, Norway; 7 Center for Muscle and Joint Health, Department of Sports Science and Clinical Biomechanics, University of Southern Denmark, Odense, Denmark; 8 Chiropractic Knowledge Hub, Odense, Denmark; 9 Helse I Hardanger, Øystese, Norway

**Keywords:** Patient activation, Work ability, Self-management strategies, Behaviour change techniques, GLA:D® Back, Concentrated treatment format, Functional recovery, Work ability, Rehabilitation adherence, PUSH project

## Abstract

**Background:**

Chronic low back pain (CLBP) is a leading cause of disability and reduced work participation worldwide. Its multifactorial nature—often lacking a clear pathological cause—poses significant challenges for effective treatment. This study reports short-term outcomes from a concentrated, interdisciplinary group-based intervention targeting patients with hard-to-treat CLBP.

**Methods:**

This study reports outcomes from the CLBP arm of a nonrandomized five-armed transdiagnostic, pre-post intervention trial (2020–2022); no control group or between-group comparisons are included. The intervention comprised SMART (Specific, Measurable, Achievable, Relevant, Time-bound) goal setting, multilevel exercise, patient education, and micro-choice strategies—small, intentional decisions aimed at enhancing functional capacity and reducing symptom-related vigilance—delivered in a concentrated group format. Outcomes were evaluated three months post-intervention and included disability (Oswestry Disability Index, ODI), pain intensity (NRS-11), work ability, medication use, sick leave status, and physical performance tests. Continuous outcomes were analysed using mixed-effects regression models to account for repeated measures, while categorical changes were assessed using symmetry tests for ordinal variables and McNemar’s test for binary variables.

**Results:**

The majority in our cohort had low educational levels, were overweight, and welfare recipients, many had multisite pain, used daily pain medication and had a history of back surgery. At follow-up, most outcomes showed statistically significant improvement. Disability decreased by 5.9 points (-18%, 95% CI: 4.1–7.7), and low back pain intensity decreased by 1.3 points (-21%, 95% CI: 0.9–1.7). Work ability increased by 0.9 points (+ 22%, 95% CI: 0.4–1.5) and sit-to-stand repetitions increased by 3.6 (+ 27%, 95% CI: 2.6–4.7). Fifteen (17%) participants transitioned from sick leave to work, and 16 (18%) discontinued pain medication.

**Conclusions:**

Patient education, structured exercise, and the concentrated micro-choice-based intervention was associated with improved pain, disability, work ability, and physical function in a hard-to-treat CLBP population. Improvements due to regression to the mean are a possibility, therefore a randomized controlled trial is warranted to confirm efficacy.

**Trial registration:**

Clinicaltrials.gov (Identifier: NCT05234281), first submitted 26. May 2021.

## Introduction

Low back pain (LBP) is the leading global cause of years lived with disability and a major contributor to work impairment and early retirement [1]. CLBP disproportionately burdens healthcare systems due to persistent symptoms and treatment resistance [2, 3]. In clinical practice, a subset of CLBP patients—those with long-standing symptoms, prior treatment failures, and prolonged sick leave—pose challenges for rehabilitation. The present study targets this hard-to-treat group, who often fall outside the scope of standard care pathways, and lack a good, tailored treatment option. Despite its prevalence, most CLBP cases lack a clear pathological cause and are considered multifactorial, involving complex interactions between biological, psychological, and social factors [2–7].

Current clinical guidelines recommend patient education, physical activity, exercise therapy, and psychosocial interventions [5, 8, 9]. While several randomized controlled trials (RCTs) have demonstrated the efficacy of behavioural interventions for CLBP [4, 10], many patients continue to experience modest and inconsistent improvements in pain and function [6, 10]. Moreover, fragmented care pathways and conflicting treatment goals may hinder recovery and increase cumulative costs [1, 3]. These challenges highlight the need for integrated, cost-effective interventions tailored to the heterogeneous nature of CLBP—particularly for individuals who do not respond adequately to standard treatments.

The PUSH project (“Prosjekt Utvikling av Smarte Helseløsninger”; Project Development of Smarter Health Solutions) was developed to address this gap. The intervention combines principles from the Bergen 4-Day Treatment (B4DT), originally developed for obsessive–compulsive disorder [11], with the structured education and exercise components of the Danish GLA:D® Back program [12]. Central to PUSH is the concept of behavioural change through “micro-choices”—small, intentional decisions that challenge symptom-driven habits and promote functional recovery. Micro-choices is a novel concept defined as subtle, repeated behavioural shifts—such as choosing to move despite discomfort or redirecting attention away from pain—that support increased behavioural flexibility. These decisions are individualized and context-specific, and are designed to counter maladaptive patterns like avoidance, hypervigilance, or excessive care-seeking. The framework is described in detail in the protocol paper by Kvale et al. [13].

Consistent with the behavioural focus of the intervention, symptom reduction was not emphasized. Instead, progress was defined by observable changes in behaviour and functional capacity. The integration of GLA:D® Back provided a complementary framework for patient education and supervised group exercise, supporting sustainable self-management strategies [12].

Although the present study uses a single-arm, quasi-experimental design without a control group, it provides valuable insight into short-term outcomes and implementation potential in a real-world clinical setting. The design was chosen to reflect the practical constraints and clinical realities of treating a complex patient population often excluded from randomized trials. A previous publication from the PUSH group reported high levels of patient satisfaction and acceptance of the four-day program [14]. Among participants with CLBP, the intervention yielded small positive effects on work and social functioning, large potential improvements in illness perception, and statistically significant gains in self-perceived health [14].

Given the chronicity and complexity of this patient group—including prior surgery, prolonged sick leave, and multiple comorbidities—achieving functional improvement is particularly challenging. Hence, this study aims to evaluate outcomes of a concentrated, interdisciplinary intervention designed to address these barriers. We hypothesized that participants undergoing the concentrated, interdisciplinary program would demonstrate clinically meaningful improvements in pain, function, and work participation compared with their baseline status.

## Methods

### Study design and participants

This pre-post intervention study used a within-subjects design, where participants served as their own controls. Baseline measurements were compared to outcomes at a three-month follow-up. The primary outcome is change in disability levels. Secondary outcomes include pain intensity, physical function, use of pain medication, sick leave status, and work ability.

The PUSH project was a collaboration between Haukeland University Hospital (HUH, Bergen, Norway) and “Helse i Hardanger” (HIH, Øystese, Norway). Although the intervention was delivered at a central location, follow-up for participants in the chronic low back pain (CLBP) arm was conducted at trained satellite clinics across three adjacent municipalities [13].

Participants were recruited between December 2020 and September 2022. Inclusion criteria required participants to be of working age (18–70 years), have CLBP as their primary diagnosis (ICD-10: M54.4/0.5, and duration ≥ 3 months), and have experienced at least four months of sick leave in the past year, the ability to complete questionnaires in Norwegian, and the capacity to participate in eight weeks of local group training during phase three (the follow-up phase) of the intervention. Important exclusion criteria included no recent surgery (within the past eight weeks).

Possible participants were originally referred to the Department of Physical Medicine and Rehabilitation at Haukeland University Hospital (HUH) by primary care providers, including general practitioners, chiropractors, and specialist physiotherapists. Screening was conducted by a chiropractor and a specialist in physical medicine and rehabilitation, using a combination of clinical examination and structured interviews. Patients who were not eligible for other established rehabilitation programs at the department (most often due to having been on sick leave for more than four months) were considered for inclusion in the PUSH LBP study. The original target sample size was 200 participants, but this was adjusted due to disruptions related to the COVID-19 pandemic. No formal power calculation was done for this trial.

### Ethics approval, data collection and -protection

The PUSH study was approved by the Regional Committee for Medical and Health Research Ethics, Western Norway (REK Vest; reference number 2020/101648), and conducted in accordance with the Declaration of Helsinki. Written informed consent to participate was obtained from all participants in the study. Data were collected digitally at two time points: baseline and three months post-intervention.

Physical assessments were conducted during the first and final group training sessions, using four standardized clinical tests of spinal flexibility, strength, and endurance, in accordance with the GLA:D® Back protocol [9]. Results were digitally reported by the assessing clinician.

All data and communication were managed through a secure, encrypted digital platform developed by YouWell (Bergen, Norway) [15]. De-identified datasets were transferred to an encrypted, access-controlled research server to ensure data privacy and integrity.

### Intervention and follow-up

In short, the PUSH project tested a concentrated micro-choice intervention across chronic conditions, including CLBP [13]. Each diagnostic group had a dedicated clinical lead and a cross-disciplinary team.

The intervention followed a three-phase model [13]:

- Phase 1 (Preparation): Addressed potential barriers to participation and introduced core concepts.
- Phase 2 (Intervention): Delivered a concentrated four-day program at HIH’s facility in Øystese, Norway.
- Phase 3 (Integration): Focused on applying learned strategies in daily life, supported by digital follow-up and group training.

For the CLBP group, the GLA:D® Back program was integrated into all three phases. This included an additional educational session in Phase 1, two one-hour group training sessions and standardized education in Phase 2, and eight weeks of twice-weekly group training at satellite clinics in Phase 3. Participants selected one of four difficulty levels for each of nine exercises. The GLA:D® Back materials and questionnaires were used in their Norwegian-translated versions [12, 16].

To ensure consistency, all procedures were documented in a standard operating procedure. The intervention was delivered by a multidisciplinary team including medical doctors, nurses, physiotherapists, chiropractors, pharmacists, and psychologists—all trained in the micro-choice based concentrated treatment format.

The framework for the micro-choice treatment is described in detail in the protocol paper [13]. The intervention was individually tailored, with participants identifying specific behavioural patterns that interfered with daily functioning. These patterns were addressed through guided micro-choice strategies, which emphasized small, actionable decisions to counter avoidance, hypervigilance, or over-reliance on care. The approach was supported by SMART goals and daily self-monitoring.

**Fig. 1:**
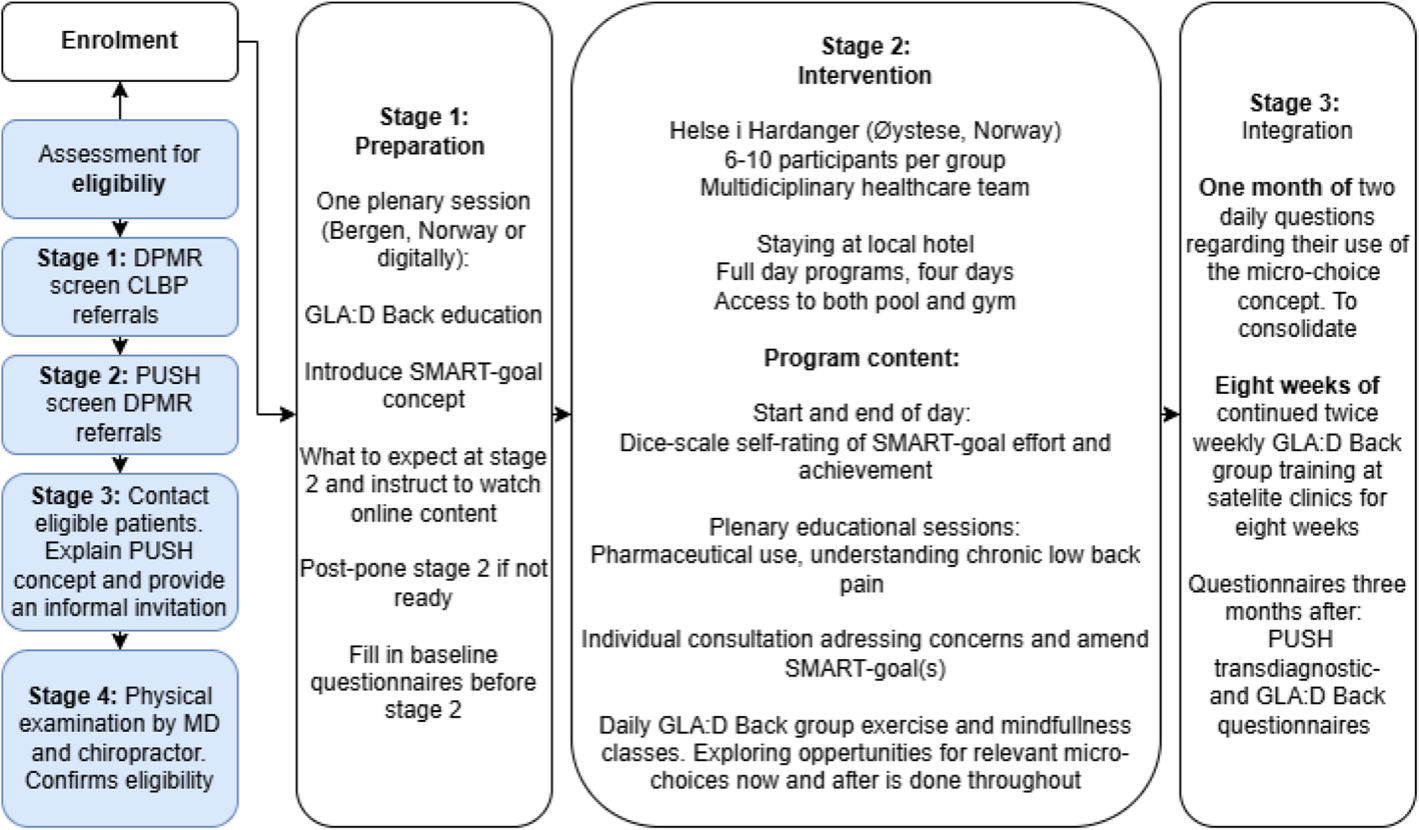
Overview of the PUSH LBP intervention and follow-up for participants with chronic low back pain. The PUSH LBP program followed a three-phase micro-choice–based treatment model integrated with the GLA:D® Back program. *Stage 1 (Preparation)* included screening, eligibility assessment, baseline questionnaires, and a plenary session introducing GLA:D® Back education and SMART-goal setting. *Stage 2 (Intervention)* consisted of a four-day concentrated program delivered by a multidisciplinary team at Helse i Hardanger, including daily group sessions, individualized consultations, and GLA:D® Back exercise and mindfulness classes. *Stage 3 (Integration)* involved one month of daily self-monitoring of micro-choice use and eight weeks of continued twice-weekly GLA:D® Back group training at satellite clinics. Follow-up questionnaires were completed three months after the intervention

 During follow-up, participants could contact the HIH team via a web application. They were encouraged to focus on one to two SMART goals (Specific, Measurable, Achievable, Relevant, Time-bound) [17–19], defined in Phase 1 and refined in Phase 2. To facilitate integration of the changes into daily life, self-reporting was encouraged during the first three weeks of Phase 3, using two visual analogue scale (VAS) items (Fig. 1):

1. The extent to which symptoms guided behaviour
2. The extent to which micro-choice strategies were applied to counter maladaptive habits

## Outcomes

### Outcome measures

The primary outcome of this study was low back pain-specific disability, assessed using the Norwegian version of the Oswestry Disability Index (ODI) [20, 21]. The ODI consists of ten items covering various aspects of functional capacity. Total scores are converted to a percentage scale ranging from 0 to 100, with higher scores indicating greater disability. Disability levels were categorized into five intervals: minimal (0–20), moderate (21–40), severe (41–60), crippled (61–80), and bedridden (81–100) [21, 22].

Secondary outcomes included both self-reported and clinician-assessed measures of pain, function, and work-related status. Functional capacity was evaluated using five numerical rating scales (NRS-11), each ranging from 0 to 10, covering endurance, strength, mobility, balance, and freedom of movement [16, 23]. Higher scores indicated better functional capacity. Pain intensity was assessed using two NRS-11 scales targeting low back pain and sciatic nerve pain, where higher scores reflected greater pain severity [16, 24]. Medication use was captured through a categorical item with four response options: prescription medication, non-prescription medication, both, or none [16]. For analysis, responses were dichotomized into “yes” and “no”. Work-related outcomes included two items: (1) current sick leave status (yes/no); and (2) current work ability, rated on an NRS-11 scale (Work Ability Index) [16, 25].

In addition to questionnaire data, participants completed four standardized physical tests designed to assess spinal flexibility, strength, and endurance in the lower back and legs [16, 26–29]. These included:

1. Forward bend from standing**:** scored across four performance levels based on range of motion and pain.
2. Curl-up hold: performed from a supine position and timed in seconds (0–120).
3. Back extension hold: performed from a prone position and timed in seconds (0–180).
4. Sit-to-stand test: number of repetitions completed in 30 s using a standard chair (max 25).

All physical tests were administered by trained clinicians (chiropractors or physiotherapists) at satellite clinics. Detailed descriptions and illustrations of these tests are available in the GLA:D® Back publications and their source papers [12, 16, 26–29].

Adverse events were reported using the following yes/no question. “Have you experienced harmful effects or developed problems because of you GLA:D Back participation?”.

If answered yes, then three possible free-text responses were available. Q1.1: Describe harmful effects that resulted in additional treatment needs, Q1.2: Describe other harmful effects, and Q1.3: Describe your side-effects.

### Statistical analysis

Descriptive statistics were used to summarize baseline characteristics.

Changes from pre- to post-intervention were analysed using mixed-effects regression models for continuous outcomes, including the Oswestry Disability Index (ODI) total score, secondary functional capacity measures, work ability, pain intensity, and three of the four physical performance tests (curl-up hold, back extension hold, and sit-to-stand). These models accounted for repeated measures within individuals. Ninety-five percent confidence intervals (CI95%) were calculated using the standard formula: mean ± 1.96 × standard error (SE), based on the model-estimated means and SEs from the mixed-effects regression models. Effect sizes were calculated using Glass’s Δ, defined as the difference between pre- and post-intervention means divided by the pre-intervention standard deviation. This approach is recommended when the intervention may affect both the mean and variability of outcomes as heteroscedasticity is anticipated in pre-post within-subject designs [30]. For interpretability, effect sizes were categorized using Cohen’s conventional thresholds: small (0.2), medium (0.5), and large (0.8) [30, 31].

Categorical changes in disability levels, based on five predefined ODI categories, were analysed using a symmetry test. This test evaluates whether the distribution of participants shifting between categories is symmetric across the diagonal of a contingency table. These k × k contingency tables also provide a responder-level view of change, illustrating individual transitions in disability severity and forward bend physical test performance.

Changes in binary outcomes, such as current use of pain medication and sick leave status, were assessed using McNemar’s test, and odds ratios (ORs) were calculated to quantify the direction and magnitude of change.

## Results

### Participant flow and inclusion

**Fig. 2:**
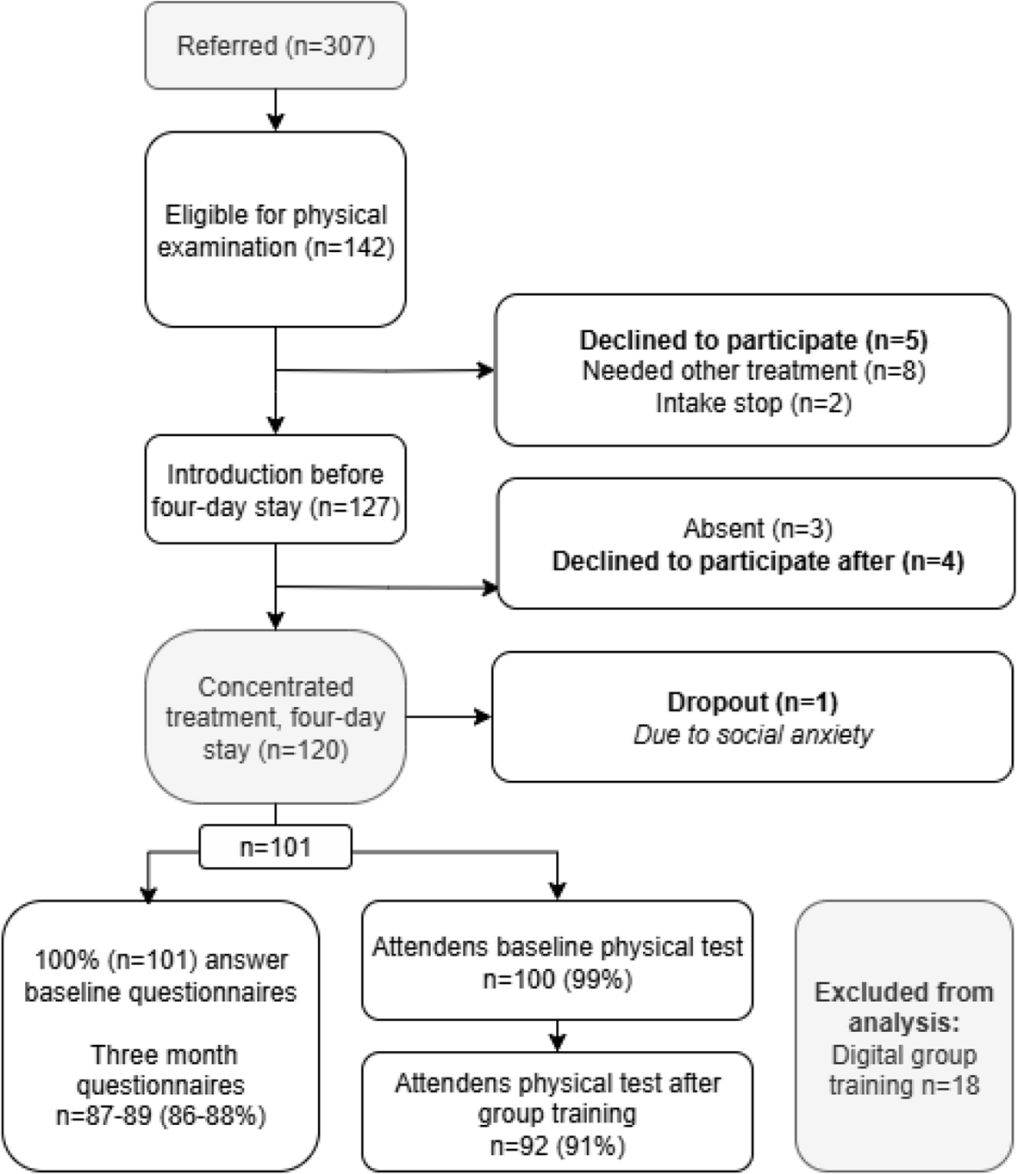
Participant flow from referral to three-month follow-up. *Declined to participate –* patients who opted out at various stages. *Excluded from analysis –* participants who received digital group training; not included in this analysis due to differences in intervention delivery. *Follow-up response rates –* number of participants who completed questionnaires or physical tests at three months. * Percentage refers to participants who completed follow-up assessments. *Dropout –* one participant who withdrew during the four-day stay due to social anxiety

Of the 132 patients assessed as eligible, 9 declined participation, 3 were absent for the introduction, resulting in 120 enrolled individuals. One participant withdrew during the intervention due to social anxiety, and 18 participants received digital group training instead of in-person sessions due to external circumstances and were therefore not included in this analysis. This yielded a final sample of 101 participants for the analysis (Fig. 2).

Overall, the group was young to middle aged with relatively low educational levels, overweight or obese, and most received welfare benefits (Table 1). The vast majority had comorbid conditions, multisite pain, pain duration of one year or more, used pain medication and had had previous back surgery (Table 1).

**Table 1:** Participant demographics at baseline

*N* = 101	N (%)/Mean (SD)
Gender	
Male	46 (45%)
Female	55 (55%)
Age	43.5 (11)
Height	175 (9)
Weight	86 (17)
BMI	28 (5)
Education	
≤ High School	57 (56%)
Higher education, ≤ 4 years	35 (35%)
Higher education, > 4 years	9 (9%)
Workstatus	
Working, no sick leave	25 (25%)
Sick leave	52 (52%)
AAP^†^	11 (11%)
Disability benefit^‡^	4 (4%)
Unemployed	8 (8%)
Retired	1 (1%)
Comorbidities	1.3 (1)
Multisite pain	
No additional pain sites	4 (4%)
≤ 3	27 (27%)
≥ 4	70 (69%)
Pain duration	
< 1 year	22 (22%)
≥ 1 year	79 (78%)
Pain medication use	
No medication use	32 (32%)
Use pain medication	69 (68%)
Back surgery	
No previous back surgery	24 (24%)
Has had previous back surgery	77 (76%)
*Comorbidities specified*	
Category	N (%/73)
Diabetes, type 2	3 (4%)
Osteoporosis	2 (3%)
Blood clot, heart/brain/other	1 (1%)
High blood pressure or other circulatory disturbance	19 (26%)
Arthrosis	14 (19%)
Rheumatic disease	4 (5%)
Metabolic disorders, high/low	7 (10%)
Asthma	17 (23%)
Migraines	22 (30%)
Stomach-/Gut-disorders	11 (15%)
Cancer	1 (1%)
COPD	3 (4%)
Sclerosis, Parkinson or other neurologic disorders	4 (5%)
Depression or anxiety	24 (33%)

^†^Work assessment allowance (AAP): Temporary benefit for individuals with ≥ 50% reduced work capacity due to illness or injury, typically granted for up to three years, with possible extension

^‡^Disability benefit: Permanent income support for individuals with ≥ 50% permanently reduced work and income capacity due to illness or injury

A significant improvement in disability was observed, with a mean reduction in ODI total score of 5.9 points (−18%, CI: 4.1–7.7), corresponding to a moderate effect size (Glass’s Δ = 0.6), yet this may not be of clinical significance as suggested thresholds are either a ≥ 10 point or a ≥ 30% reduction for the ODI scale (Table 2) [32, 33]. Among the 87 participants with complete data, 24 (28%) transitioned from moderate to minimal disability, while only 4 (5%) showed the opposite pattern (Table 3). The contingency table for ODI categories illustrates individual-level transitions in disability severity and can be interpreted as a form of responder analysis.

**Table 2:** Estimated changes in functional capacity, pain, and physical performance from baseline to three-month follow-up

	**Baseline**	**3 months**			
	***M* (*SE*) [CI95%]**	***M* (*SE*) [CI95%]**	***Z***	***p***	**Glass’s Δ**
Functional capacity					
ODI total score	31.9 (1.1) [29.7, 34]	26 (1.2) [23.8, 28.3]	−4.9	<.001	0.6
Strength	4.3 (0.2) [3.9, 4.6]	5.1 (0.2) [4.7, 5.5]	4.7	<.001	0.4
Endurance	4 (0.2) [3.6, 4.4]	4.8 (0.2) [4.3, 5.2]	3.7	<.001	0.4
Mobility	3.9 (0.2) [3.4, 4.3]	4.5 (0.2) [4, 4.9]	2.9	<.01	0.3
Balance	4.4 (0.2) [4, 4.9]	5.1 (0.2) [4.7, 5.6]	3.7	<.001	0.3
Freedom of movement	4.2 (0.2) [3.9, 4.6]	5.3 (0.2) [4.9, 5.7]	4.9	<.001	0.6
Work ability	4.2 (0.3) [3.7, 4.7]	5.1 (0.3) [4.6, 5.7]	3.4	<.01	0.4
Pain measures					
LPB	6.1 (0.2) [5.8, 6.5]	4.8 (0.2) [4.4, 5.2]	−5.8	<.001	0.7
Sciatica	3.9 (0.3) [3.4, 4.5]	3.5 (0.3) [2.9, 4.1]	−1.2	.3	0.1
Physical test performance					
Curl-up	59.2 (3.6) [52.2, 66.2]	76.6 (3.6) [69.4, 83.7]	6	<.001	0.5
Back-raise	107.8 (5.1) [97.8, 117.8]	131.7 (5.3) [121.4, 142]	5.1	<.001	0.5
Sit-to-stand	13.56 (0.6) [12.3, 14.8]	17.2 (0.7) [15.9, 18.5]	8.7	<.001	0.7

Estimated means with standard errors (SE), 95% confidence intervals (CI), Z-scores, p-values, and effect sizes (Glass’s Δ) for changes from baseline to follow-up. Outcomes include the Oswestry Disability Index (ODI) total score, secondary functional capacity measures (strength, endurance, mobility, balance, freedom of movement, and work ability), low back pain, sciatica, and physical performance tests (curl-up hold, back extension hold, and sit-to-stand). *n* = 101

**Table 3:** Contingency table, ODI

		ODI categories at follow-up*					**Total**
		1	2	3	4	﻿5	
ODI categories at baseline*	1	9 (*n*_11_)	4 (*n*_12_)	0 (*n*_13_)	0 (*n*_14_)	0 (*n*_15_)	13
	2	24 (*n*_21_)	29 (*n*_22_)	4 (*n*_23_)	0 (*n*_24_)	0 (*n*_25_)	57
	3	3 (*n*_31_)	5 (*n*_32_)	8 (*n*_33_)	1 (*n*_34_)	0 (*n*_35_)	17
	4	0 (*n*_41_)	0 (*n*_42_)	0 (*n*_43_)	0 (*n*_44_)	0 (*n*_45_)	0
	5	0 (*n*_51_)	0 (*n*_52_)	0 (*n*_53_)	0 (*n*_54_)	0 (*n*_55_)	0
	Total	36	38	12	1	0	87

Contingency table of ODI categories at pre- and post-treatment. Symmetry test of equality of corresponding cell proportions, symmetry Χ^2^ (4) = 18.40, *p* <.001. *n* = 87

^*^ ODI categories are as follows: 1 = Minimal disability; 2 = Moderate disability; 3 = Severe disability; 4 = Crippled; 5 = Bed-bound. Inspecting the off-diagonal cell pairs reveals that cells n_21_ and n_12_ contributed most to the symmetry Χ^2^ (14.29), corresponding to changes between categories 1 (“minimal disability) and 2 (“moderate disability”)

All secondary functional capacity measures showed significant improvements with moderate effect sizes, with mean differences ranging from + 0.6 to + 1 points. Though possibly not clinically significant as NRS-11 scales usually demand an improvement of ≥ 2 points or ≥ 30% for a clinically significant improvement [32, 33]. Strength improved by 0.8 points (+ 19%, 95% CI: 0.4–1.2, Δ = 0.4), endurance by 0.8 points (+ 20%, 95% CI: 0.3–1.3, Δ = 0.4), mobility by 0.6 points (+ 16%, 95% CI: 0.2–1, Δ = 0.3), balance by 0.7 points (+ 16%, 95% CI: 0.3–1.1, Δ = 0.3), and freedom of movement by 1 points (+ 25%, 95% CI: 0.7–1.4, Δ = 0.6).

Work ability increased by 0.9 points (+ 22%, 95% CI: 0.4–1.5, Δ = 0.4). Of 89 participants, 15 returned to work and 2 went on sick leave—a net absolute improvement for 13 (15%). To illustrate the direction and magnitude of change, we calculated an odds ratio of 7.5, indicating that participants were substantially more likely to discontinue sick leave than to initiate it.

Low back pain decreased by 1.3 points (−21%, 95% CI: 0.9–1.7), corresponding to a moderate effect size (Δ = 0.7). No significant change was observed for sciatica.

All three physical performance tests showed significant improvements with moderate effect sizes. Curl-up hold increased by 17.4 s (+ 29%, 95% CI: 14.0–20.8, Δ = 0.5), back-raise hold by 23.9 s (+ 22%, 95% CI: 18.6–29.2, Δ = 0.5), and sit-to-stand repetitions by 3.6 (+ 27%, 95% CI: 2.6–4.7, Δ = 0.7). In the forward bend test, 31 participants (34%) improved from restricted movement with pain to full mobility (with or without pain), while only one participant showed deterioration. An additional 7 participants (7.7%) improved from painless restriction to free movement without pain (Table 4).

**Table 4:** Contingency table, forward bend test

		Forward-bend at post-treatment*				**Total**
		1	2	3	4	
Forward-bend at pre-treatment*	1	11 (*n*_11_)	0 (*n*_12_)	16 (*n*_13_)	16 (*n*_14_)	42
	2	0 (*n*_21_)	2 (*n*_22_)	2 (*n*_23_)	7 (*n*_24_)	11
	3	1 (*n*_31_)	2 (*n*_32_)	2 (*n*_33_)	8 (*n*_34_)	13
	4	0 (*n*_41_)	0 (*n*_42_)	4 (*n*_43_)	21 (*n*_44_)	25
	Total	12	4	23	52	91

Symmetry test of equality of corresponding cell proportions in the forward-bend physical test, symmetry Χ^2^ (3) = 36.6, *p* <.001. *n* = 91

^*^Categories for the physical test are as follows: 1 = test completed with pain and without normal freedom of movement; 2 = test completed without pain, but not normal freedom of movement; 3 = test completed with pain and with normal freedom of movement; 4 = test completed without pain and with normal freedom of movement. Inspecting the off-diagonal cell pairs revealed that cells n_14_ and n_41_ contributed most to the symmetry Χ^2^ (16), followed by cells n_13_/n_31_ (Χ^2^ = 12.3) and cells n_24_/n_42_ (Χ^2^ = 7)

Among 87 participants with complete data, 16 reported discontinuing pain medication use from baseline to follow-up, while 6 began using it. This corresponds to a net cessation of medication use for 10 participants (11%). The odds ratio of 2.7 suggests that participants were more likely to stop using medication than to start.

Adverse events were reported by 16 participants (16%). When bulked together free text answers show that three participants experienced worsening of their sciatica, of which one had to start using stronger pain medication, and one had a new MRI resulting in a surgical intervention due to lateral canal stenosis. The remaining 13 either fail to answer the free text questions (*n* = 5), or report increased low back-, knee- or buttocks pain.

## Discussion

This study demonstrates that a concentrated, micro-choice-based intervention combined with GLA:D® Back may be associated with clinically relevant within-subject short-term improvements in disability, pain intensity, physical performance, work ability, sick leave status, and pain medication use. Two prominent changes were a 5.9-point (18%) reduction in ODI scores and a 1.3-point (21%) reduction in low back pain intensity, both corresponding to moderate effect sizes and supported by narrow confidence intervals.

Although no covariate adjustments were made these findings are particularly compelling given the poor prognostic profile of the study population. Participants commonly presented with multiple prognostic risk factors, including low educational attainment, obesity, comorbidities, long-standing and multisite pain, welfare dependency, and prior back surgery—all of which are associated with poorer outcomes in chronic low back pain [2, 6, 7, 34–39]. Despite these challenges, the intervention was associated with outcomes that are comparable to, or exceed, those typically reported in multidisciplinary biopsychosocial rehabilitation (MBR) programs. In such programs, a large effect size (≈0.7) is commonly observed for improvements in low back pain intensity, and a moderate effect (≈0.5) for disability [9, 10]. The high follow-up completion rate (90%) further strengthens the validity of the findings and may reflect the structured nature of the intervention and the completeness of baseline data [13, 40]. While micro-choice strategies may enhance autonomy and behavioural flexibility, they may be less suitable for patients with low readiness for change or limited cognitive/psychological resources, and require a high level of clinician competence.

 The concept of minimal clinically important difference (MCID) is complex and context-sensitive, influenced by population characteristics, intervention type, and analytic approach [32, 33, 41]. Although the commonly cited ≥ 30% threshold was not met at the group level, approximately 25% of participants achieved this level of improvement individually. This highlights the value of responder-level analysis in heterogeneous, treatment-resistant populations. In such cohorts, even modest gains—especially when supported by functional improvements—may represent meaningful clinical change. The use of k × k contingency tables for ODI adds granularity to this interpretation by illustrating individual transitions in disability severity.

 The possible improvements in physical performance may have been associated with functional gains and reduced disability. Given the group’s complex clinical profile—including long-standing symptoms, prior surgery, and ongoing compensation due to illness or injury—even small gains may be clinically relevant. The observed gains in spinal flexibility and muscular endurance, as measured by standardized physical tests, support the intervention’s impact on physical function.

The discontinuation of pain medication by several participants could reflect not only reduced pain intensity but also enhanced coping strategies, potentially fostered by the behavioural focus of the intervention. Positive changes in sick leave status and self-reported work ability may be associated with the interventional impact on functional recovery and social participation [14]. These outcomes align with the intervention’s emphasis on guided self-care and SMART goal setting, which may have empowered participants to re-engage with work and daily activities.

Compared to the broader patient population at the referral institution (NNRR), PUSH LBP participants had lower educational levels, higher rates of prior surgery, longer pain duration, and greater welfare dependency [42, 43]. Despite these differences, the intervention was associated with similar reductions in pain and disability, suggesting its potential as an alternative care model.

The emphasis on participant-led SMART goals and guided self-care aligns with emerging evidence supporting empowerment-based approaches in chronic pain management [44–47]. While outcomes in our study were somewhat inferior to those reported in Cognitive Functional Therapy (CFT) trials, differences in study design (RCT vs. quasi-experimental), population characteristics, and intervention intensity limit direct comparisons. Therefore, comparisons with CFT and MBR trials should be interpreted cautiously, as these interventions differ in structure, delivery format, and the clinical context in which they are applied. Nonetheless, the observed pain intensity decrease—particularly among participants with prior surgery—fall within the range reported in CFT studies and suggest potential relevance for similar subgroups [48–50].

### Methodological considerations

Several limitations must be acknowledged, and these can be grouped into three main categories: design-related, measurement-related, and contextual factors.

Design-related limitations include the single-arm pre–post design, which restricts causal inference and increases susceptibility to confounding from time-related or external factors [51]. Without a control group, it is difficult to determine whether observed possible improvements stem from the intervention itself or from other influences such as regression to the mean, spontaneous recovery, or participant expectations. While regression to the mean cannot be ruled out, the consistency and magnitude of improvements across multiple domains suggest that the observed changes may reflect more than statistical artefact. These findings warrant confirmation in randomized controlled trials that can more rigorously isolate the intervention’s impact. Additionally, the exclusion of participants who received digital group training may introduce selection bias. Differences in delivery format could influence engagement and outcomes, and future studies should consider evaluating digital versus in-person formats to assess equivalence. Additionally, we did not adjust for potential confounders such as age, medication use, or adherence, which may have influenced outcomes.

Measurement-related limitations include the extensive questionnaire burden, which may have led to respondent fatigue and affected data quality [52]. Future studies may benefit from streamlining follow-up assessments or using adaptive survey techniques to reduce burden and improve response accuracy. The Norwegian version of the GLA:D® Back questionnaires were used throughout the intervention. However, the translation process did not follow formal forward–backward translation procedures. While the materials were adapted by clinicians fluent in both languages and reviewed for clinical relevance, the absence of standardized linguistic validation may introduce subtle cultural or semantic biases. Future studies should consider formally validated translations to ensure cross-cultural comparability and measurement precision. Additionally, outcome assessors were not blinded, which may have introduced bias in clinician-administered physical tests.

Contextual limitations include potential misunderstandings in self-reported sick leave status due to complexities in Norway’s welfare system, particularly the AAP period, which blends sick leave with mandatory work training. The COVID-19 pandemic may also have influenced recruitment and sample diversity, although similar interventions in Denmark were reportedly unaffected [52]. Although clinicians followed a standardized protocol, subtle differences in communication style, emphasis, or group cohesion may have influenced participant engagement and outcomes—factors not captured in this analysis [53].

Despite these limitations, the study’s inclusive design enhances generalizability. Many intervention studies exclude individuals with poor prognostic indicators, yet these individuals represent a substantial portion of real-world clinical populations [6]. Thus, while findings may not generalize to all CLBP populations, they are likely applicable to similar high-need cohorts characterized by chronicity, prior treatment failure, and welfare dependency. The use of mixed-effects regression models allowed for robust handling of repeated measures and missing data, further strengthening the internal validity of the findings.

## Conclusion

In this exploratory evaluation of a concentrated micro-choice based intervention for chronic low back pain, possible short-term improvements were observed in back-related disability, pain intensity, work ability, physical performance, pain medication use, and sick leave status. The intervention may yield meaningful improvements in hard-to-treat CLBP, although causal inference is limited by the study design. While a randomized controlled trial would be ideal to confirm efficacy, such a study may be challenging to implement in this population. Nonetheless, the present findings offer a foundation for future comparative research and suggest that concentrated, behaviourally focused interventions may offer meaningful benefits for treatment-resistant CLBP populations. Future research should also explore long-term outcomes and assess changes in work status and healthcare utilization using national registry data.

## Data Availability

For access to the data and materials used in this study, please contact the corresponding author. Requests will be forwarded to YouWell and the data ownership group for consideration and approval.

## Abbreviations

AAP: Work assessment allowance
B4DT: Bergen 4-day treatment, an innovative treatment for obsessive–compulsive disorders
CFT: Cognitive functional therapy
CLBP: Chronic low back pain
DPMR: Department of physical medicine and rehabilitation
GLA:D® Back: A standardized care package for chronic low back pain patients, developed in Denmark
HIH: Helse I Hardanger, a healthcare provider previously based in Øystese, Norway
HUH: Haukeland university hospital, the regional hospital
LBP: Low back pain
MBR: Multidisciplinary biopsychosocial rehabilitation
MCID: Minimal clinically important difference
NNRR: Norwegian neck- and back registry
NRS-11: Numerical rating scale, typically ranging from 0 to 10
ODI: Oswestry disability index
PUSH: Prosjekt utvikling av smarte helseløsninger (Norwegian) / Project development of smarter healthcare solutions (English)
REK: Regional ethics committee
SMART: Specific, measurable, achievable, relevant, and time-bound. A format used for goal setting
VAS: Visual analogue scale
